# Effects of Two Melt Extrusion Based Additive Manufacturing Technologies and Common Sterilization Methods on the Properties of a Medical Grade PLGA Copolymer

**DOI:** 10.3390/polym13040572

**Published:** 2021-02-14

**Authors:** Marion Gradwohl, Feng Chai, Julien Payen, Pierre Guerreschi, Philippe Marchetti, Nicolas Blanchemain

**Affiliations:** 1 U1008 Controlled Drug Delivery Systems and Biomaterials, Institut National de la Santé et de la Recherche Médicale (INSERM), Centre Hospitalier Régional Universitaire de Lille (CHU Lille), University of Lille, F-59000 Lille, France; marion.gradwohl@gmail.com (M.G.); feng.hildebrand@univ-lille.fr (F.C.); pierre.guerreschi@chru-lille.fr (P.G.); 2 UMR 9020–UMR-S 1277–Canther–Cancer Heterogeneity, Plasticity and Resistance to Therapies, Institut de Recherche contre le Cancer de Lille, University Lille, CNRS, Inserm, CHU Lille, F-59000 Lille, France; philippe.marchetti@inserm.fr; 3 LATTICE MEDICAL, F-59120 Loos, France; julien.payen@lattice-medical.com; 4 Service de Chirurgie Plastique Reconstructrice et Esthétique, CHU de Lille, F-59037 Lille, France; 5 Banque de Tissus, Centre de Biologie-Pathologie, CHU Lille, F-59000 Lille, France

**Keywords:** additive manufacturing, sterilization, medical devices, bioabsorbable, polymer

## Abstract

Although bioabsorbable polymers have garnered increasing attention because of their potential in tissue engineering applications, to our knowledge there are only a few bioabsorbable 3D printed medical devices on the market thus far. In this study, we assessed the processability of medical grade Poly(lactic-*co*-glycolic) Acid (PLGA)85:15 via two additive manufacturing technologies: Fused Filament Fabrication (FFF) and Direct Pellet Printing (DPP) to highlight the least destructive technology towards PLGA. To quantify PLGA degradation, its molecular weight (gel permeation chromatography (GPC)) as well as its thermal properties (differential scanning calorimetry (DSC)) were evaluated at each processing step, including sterilization with conventional methods (ethylene oxide, gamma, and beta irradiation). Results show that 3D printing of PLGA on a DPP printer significantly decreased the number-average molecular weight (*M*_n_) to the greatest extent (26% *M*_n_ loss, *p* < 0.0001) as it applies a longer residence time and higher shear stress compared to classic FFF (19% *M*_n_ loss, *p* < 0.0001). Among all sterilization methods tested, ethylene oxide seems to be the most appropriate, as it leads to no significant changes in PLGA properties. After sterilization, all samples were considered to be non-toxic, as cell viability was above 70% compared to the control, indicating that this manufacturing route could be used for the development of bioabsorbable medical devices. Based on our observations, we recommend using FFF printing and ethylene oxide sterilization to produce PLGA medical devices.

## 1. Introduction

In recent years, additive manufacturing (AM), also known as 3D printing, has penetrated the healthcare industry, as it enables producing patient specific implants that are customized to the patient’s anatomy using complex geometry. Among AM technologies, melt-extrusion based methods are notably attractive, as they do not require an additional crosslinker or solvents which could lead to a biocompatibility issue. AM was initially used for prototyping; however, it has rapidly become a real production tool because it allows reducing costs and product development times compared to traditional processing methods such as injection molding [1].

Bioabsorbable polymers—including polylactid acid, polyglycolid acid, polycaprolactone, and their copolymers—have been widely used for the manufacturing of medical devices such as orthopedics screws, plate, sutures, or absorbable stents [2,3,4,5,6]. As they are fully absorbed by the body and present excellent biocompatibility properties, they have generated immense interest for the manufacturing of scaffolds for tissue engineering and regenerative applications [7]. Indeed, using a bioabsorbable polymer improves patient post-healing, as it avoids needing a second intervention to remove the implant [8,9,10].

Poly(lactic-*co*-glycolic) Acid (PLGA) has been extremely commonly used in biomedical and pharmaceutical applications as a polymer. Its tunable degradation rate and mechanical characteristics can be obtained by playing with its lactide/glycolide ratio to fit desirable properties [4]. PLGA degrades mainly by hydrolysis of its ester bonds in physiological conditions, causing random chain scission [11]. Hydrolytic degradation of PLGA is known to be autocatalytic and the presence of acidic bioproducts may accelerate its degradation rate [12]. Thanks to its melt behavior and thermal properties, PLGA can be processed via melt-extrusion based additive manufacturing technologies such as fused filament fabrication (FFF) and direct extrusion-based 3D printing (DPP) [13]. Fused filament fabrication is the most commonly used AM technology where a filament is melted into a heating nozzle and deposed layer-by-layer to form the 3D construct. This process requires the manufacturing of a filament in an early stage at high temperatures and by applying mechanical stress that can alter the polymer properties before printing [14]. Direct extrusion-based 3D printing using pneumatic or screw extrusion-based systems enables using polymer pellets as a raw material and hence avoids one manufacturing step compared to FFF [15].

Polymer-based implantable medical devices should be sterilized to eliminate any living microorganisms to prevent infections. The sterilization method must be chosen in order to be the least destructive to the polymer properties and implant morphology. Moist heat is one of the most widely used sterilization methods, as it presents the advantages of being fast, simple, effective and absent of any toxic residues [16,17]. Nevertheless, the high temperatures and humidity required to eliminate viable microorganisms causes excessive degradation of the polymer material that makes it unsuitable for the sterilization of bioabsorbable polymers such as PLGA [18]. Ethylene oxide (EO) is the sterilization technique generally used for polymer-based medical devices, as it can be performed at low-temperatures, which leads to minimal changes in molecular weight compared to other conventional methods [19,20,21]. The major concern with EO sterilization is the presence of toxic residues that can reside on the implant after the process [22,23,24]. In order to remove EO residues such as ethylene glycol and ethylene hydrochloride, an aeration of EO sterilized medical devices must be performed which leads to a lengthening of the final process [25,26]. The Food and Drug Administration (FDA) has recently opened an innovation challenge to identify new sterilization techniques as alternatives to EO and this sterilization technique therefore might disappear in the next few decades [27]. Irradiation sterilization methods such as gamma or beta radiation also offer the possibility to sterilize at low temperatures [16,18,19]. Moreover, these methods are rapid and effective but are known to result in changes in material properties such as the molecular weight [28,29,30].

When dealing with the medical device industry, manufacturing process control has to be taken into consideration to ensure safety and performance of products [31]. Impacts of the whole manufacturing process must be evaluated and controlled to ensure reliable and reproductible clinical performance of devices. As a matter of fact, additive manufacturing of bioabsorbable polymer and sterilization of the final products can significantly impact the polymer features, so the whole process must be chosen in order to reduce changes in the physico-chemical properties of materials.

In this work, we assessed the impact of the full additive manufacturing route from medical-grade PLGA granules to sterile 3D constructs by comparing FFF and DPP technologies. A focus has been made on determining the impact of the whole manufacturing process by evaluating changes in crystallinity, thermal properties, and molecular weight. Different sterilization techniques such as EO, gamma radiation, and beta radiation were also investigated to determine the most appropriate method for sterilizing PLGA.

## 2. Materials and Methods

### 2.1. Material

Medical Grade 85:15 poly(l-lactide-*co*-glycolide) PURASORAB PLG 8523 (inherent viscosity 2,3 dl/g) was obtained from PURAC (CORBION, Gorinchem, The Netherlands). PLGA pellets were stored in a freezer at −15 degrees to minimize degradation and were dried at 40 °C before processing.

### 2.2. Filament and Scaffold Fabrication

**Figure 1:**
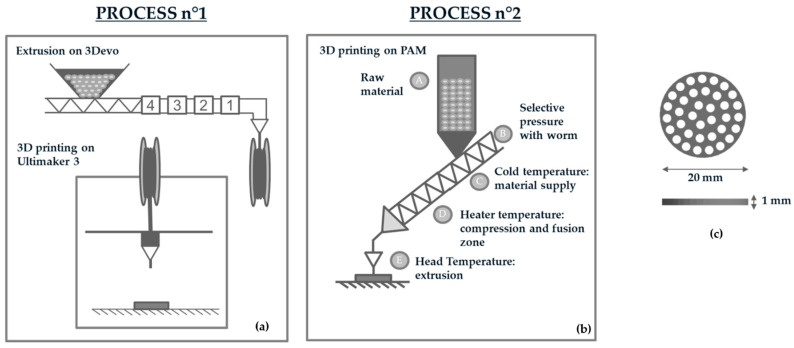
(**a**) Description of the manufacturing process n°1, including extrusion of the Poly(Lactide-*co*-Glycolide) (PLGA) filament and Fused Filament Fabrication; (**b**) Description of the manufacturing process n°2: Direct Pellet Printing; (**c**) Dimensions of the 3D printed sample.

Circular porous disc of 20 (diameter) × 1 (width) mm were designed using Solidworks Software (Figure 1c) and fabricated via FFF and DPP printing technologies for further experiments. For both printers, a nozzle of 0.40 mm was chosen to produce bioabsorbable samples.

SFor Fused Filament Fabrication (Process n°1, Figure 1a), PLGA 85:15 was first melt-spun into 2.85 mm filaments using a Composer Series 350 extruder (3DEVO, Utrecht, The Netherlands). PLGA was extruded with a speed of 7.4 rpm and the filaments diameter was controlled with an optical sensor with a precision of +/− 0.05 mm. PLGA filament was thereafter used to manufacture samples on an Ultimaker 3 printer (Ultimaker, Utrecht, The Netherlands).

A PAM (Pellet Additive Manufacturing) printer from POLLEN (Ivry-sur-Seine, France) was used in this study as a DPP printer (Process n°2, Figure 1b). This technology used pellets as a raw material and the polymer was heated progressively thanks to three heating zones: a cold extruder, main extruder, and extruder head (Figure 1b). The machine has an extrusion system inspired by the principles of an injection molding machine. A heated cylinder (zone C, Figure 1), fitted with a proprietary extrusion screw (A, Figure 1b), deposits the material evenly. Unlike standard FFF printers, the print bed moves to allow the part to be manufactured. Extrusion and printing profiles are shown in Table 1, where only parameters that may affect PLGA properties are presented (temperature, speed, flow).

### 2.3. Scaffold Sterilization

Samples were first packed in TYVEK sterilization pouches (STERICLIN, Feuchtwangen, Germany) and sent for sterilization by ethylene oxide (STERYLENE, Civrieux d’Azergues, France), gamma radiation, and beta radiation (IONISOS, Dagneux, France). The EO oxide sterilization treatment cycle was not validated according to ISO 1135 certification. Briefly, samples were first preconditioned in a separate chamber and were thereafter exposed to EO for 2 h. An aeration was then performed during 48 h to remove ethylene oxide residue from samples. For both gamma and beta sterilization, three radiation doses were evaluated: 15 kGy, 25 kGy, and 50 kGy. Gamma and beta sterilization treatment cycles were not validated according to ISO 1137 certification.

### 2.4. DSC

The thermal properties of PLGA at each step of the manufacturing process were determined by differential scanning calorimetry (DSC) under a nitrogen atmosphere with a Mettler Toledo DSC 1 apparatus (Greifensee, Switzerland). Approximatively 10 mg of samples were placed in individually aluminum pans and were heated from 20 °C to 200 °C with a heating rate of 10 °C min^−1^. The Glass Transition temperature (*T*_g_) was taken as the midpoint temperature and the melting temperature as the maximum of the endothermic peak; all the data were taken from the first heating run. Crystallinity was calculated according to the following equation: % Cristallinity= Δ*H*_m_/Δ*H*_m_°, where Δ*H*_m_ is the melting enthalpy of the sample determined from DSC and Δ*H*_m_° is the enthalpy of melting for 100% crystalline PLA 93 J·g^−1^ [32].

### 2.5. TGA

Thermogravimetric Analysis (TGA) was carried out on pellets to determine the degradation temperature of PLGA. The degradation temperature was defined as the temperature where PLGA has lost 5% of its initial mass. TGA analysis was used to define the temperature limit to avoid material degradation and therefore helped to define the appropriate printing temperature. TGA measurements were performed with a TGA Q50 apparatus (TA Instruments, New Castle, DE, USA) under nitrogen from 20 to 400 °C with a 10 °C/min heating rate.

### 2.6. Gel Permeation Chromatography (GPC)

Molecular weight distribution and weight-average (*M*_w_) and number-average molecular weight (*M*_n_) of PLGA were evaluated using a WATERS E2695 separations module system (Waters Corp., Milford, MA, USA) equipped with a Wyatt T-REX refractive index detector (Wyat Technology, Santa Barbara, CA, USA) and three STYRAGEL columns (HR1, HR3, and HR4) calibrated with polystyrene standards. Samples (30 mg) were first dissolved in Chloroform for 3 h and then dissolved in THF. Each sample was filtered through a 0.45 µm PTFE filter. Samples were eluted with THF with a flow rate of 1.0 mL·min^−1^. Each sample was run in triplicate, and the data were reported as averages.

### 2.7. Cytotoxicity Assay

The cytotoxicity of samples was further evaluated using the extraction method (ISO 10993-5), with the NIH3T3 mouse embryo Fibroblast (ATCC® CRL-1658™) by LGC Standards SARL, Molsheim, France. Sample extracts were prepared under sterile conditions by adding 200 mg into 1 mL of MEM–α culture medium supplemented with 10% fetal bovine serum (FBS, Gibco®, Thermo Fisher Scientific, Illkirch-Graffenstaden, France), which was incubated at 37 °C and with agitation at 80 rpm for 72 h to reach the requirements of the FDA for an implantable medical device. The complete culture medium was also incubated under the same conditions as the negative control. On the same day, NIH3T3 cells were plated at 4 × 103 cells/well in a 96−well tissue culture polystyrenes plate and grown in a 100 µL/well MEM–α medium supplement with 10% FBS at 37 °C and 5% CO_2_ for 24 h. The 96-well plate was partitioned into columns: the culture medium only (no cells); cells incubated in the culture medium (control); and cells incubated in an extraction medium. The medium for the monolayer cell culture was then replaced by the 100 μL/well sterile original sample extracts (filtered through a 0.2 μm sterile syringe filter) or the control medium. After 24 h of exposure of the cells to the sample extracts or control at 37 °C and 5% CO_2_, the cell viability was determined using AlamarBlue® (Gibco®, Thermo Fisher Scientific, Illkirch-Graffenstaden, France) assay. Briefly, the medium in each well was replaced with a 10% AlamarBlue® solution in the culture medium (200 μL/well), and the plate was incubated at 37 °C and 5% CO_2_ for 2 h. One hundred and fifty microliters of reacted solution per well were transferred into a 96-well plate (Fluoro–LumiNunc™, ThermoScientific, Illkirch-Graffenstaden, France). The intensity of fluorescence was determined using a Twinkle LB 970 Microplate Fluorometer (Berthold, Bad Wildbad, Germany) with an excitation wavelength of 530 nm and an emission wavelength of 590 nm. The cell survival rate was expressed by the percentage of cell viability with respect to the value of the control.

## 3. Results

### 3.1. Repetability and Printing Quality

**Figure 2:**
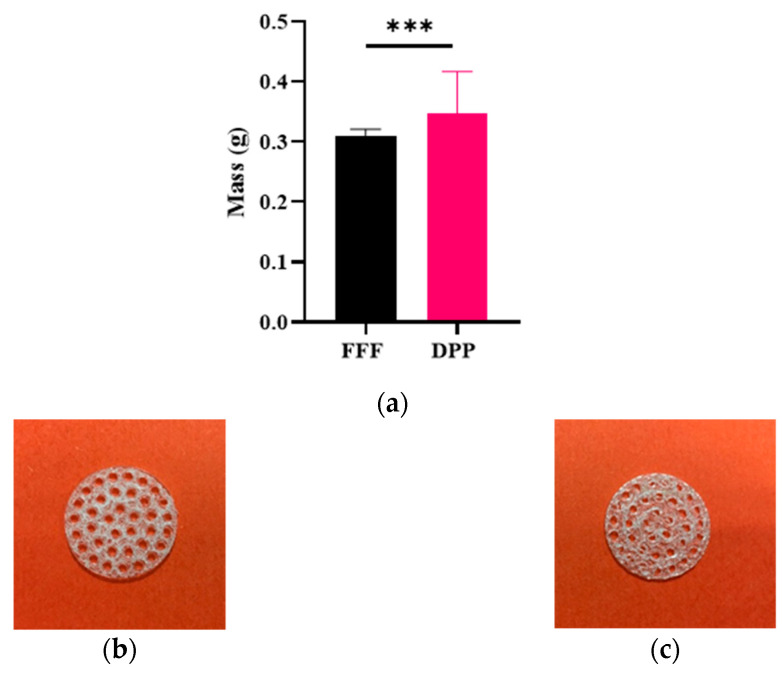
(**a**) Comparison of mass of PLGA samples printed on FFF and PAM printer. All data were analyzed using a non parametric student *t*-test *** *p* < 0.001, (**b**) FFF and (**c**) DPP printed samples.

PLGA samples were successively printed for direct pellet printing (DPP) and fused filament fabrication (FFF). However, due to melt behavior of PLGA in DPP extrusion process (Figure 2c), it was more difficult to obtain repeatability compared to for the conventional FFF process (Figure 2c). Printing profile parameters such as temperature or flow were always slightly adjusted to avoid under extrusion issues, whereas with FFF the same printing profile was used for the whole study. Moreover, this can be observed by dispersity of weight control results after 3D printing (Figure 2): DPP printed 3D samples reveals high variability (*m*_min_ = 0.204 g, *m*_max_ = 0.486 g, mean = 0.346 ± 0.07 g, *n* = 33) whereas mass values of FFF printed ones were less dispersed (*m*_min_ = 0.284 g, *m*_max_ = 0.333 g, mean= 0.311 ± 0.01 g, *n* = 56).

**Figure 3:**
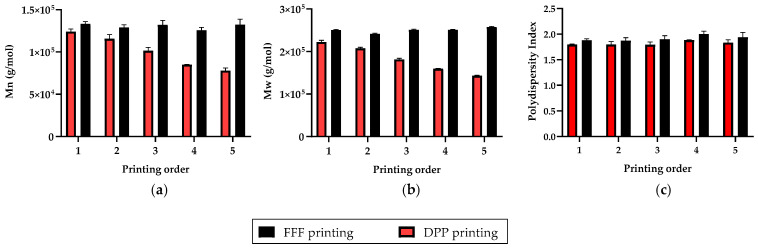
(**a**) Evaluation of (**a**) the number-average molecular weight (*M*_n_), (**b**) weight-average (*M*_w_), and (**c**) polydispersity index (IP) of PLGA 85:15 from successively printed samples; All data are expressed as mean ± SD (*n* = 3).

Repeatability of the two additive manufacturing process was investigated by successively printing five PLGA circular discs and by evaluating their molecular weight. For each sample, molecular weight analysis was performed at three different positions, and data were represented as the mean and standard deviation of these values (Figure 3).

Firstly, the final molecular weight of PLGA was lower for DPP printed samples than for FFF samples. Secondly, PLGA molecular weight of 3D perforated discs seemed to be relatively constant after successive printing, whereas for DPP the molecular weight of PLGA decreased after each print. This molecular weight decrease during successive print could be explained by the longer and uncontrolled residence time of PLGA at elevated temperatures in the extrusion mechanism of a DPP printer.

### 3.2. Impact of the Manufacturing Process

#### 3.2.1. Molecular Weight

**Figure 4:**
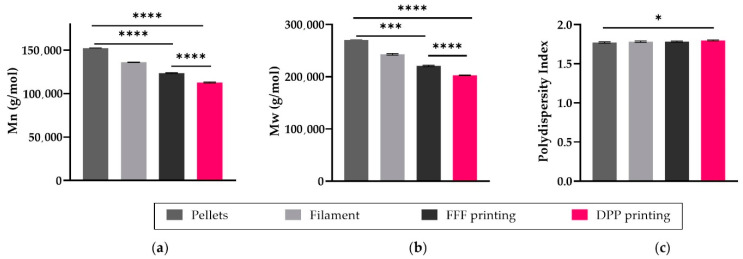
(**a**) Impact of additive manufacturing steps on the (**a**) *M*_n_, (**b**) *M*_w_ and (**c**) polydispersity index of PLGA 85:15; All data are expressed as mean ± SD (*n* = 3) and were analyzed using Anova one-way test and compared to the control * *p* < 0.05, *** *p* < 0.001, **** *p* < 0.0001.

Molecular weight analysis was performed by GPC to evaluate the impact of each step of the manufacturing process on the degradation of PLGA 85:15 and to evaluate the less impacting process between FFF and DPP (Figure 4). The weight average molecular weight and average molecular weight of PLGA decreased to about 10% of the original value after the filament-extrusion process. Size exclusion chromatography profiles indicated a decrease respectively by 19% and 26% of the PLGA *M*_n_ from 1.53 × 10^5^ g/mol for pristine material to 1.24 × 10^5^ g/mol after process n°1 and 1.13 × 10^5^ g/mol for process n°2. Even though Fused Filament Fabrication requires two thermal steps, its molecular weight drop is less important than for the DPP printer.

#### 3.2.2. Thermal Analysis

**Figure 5:**
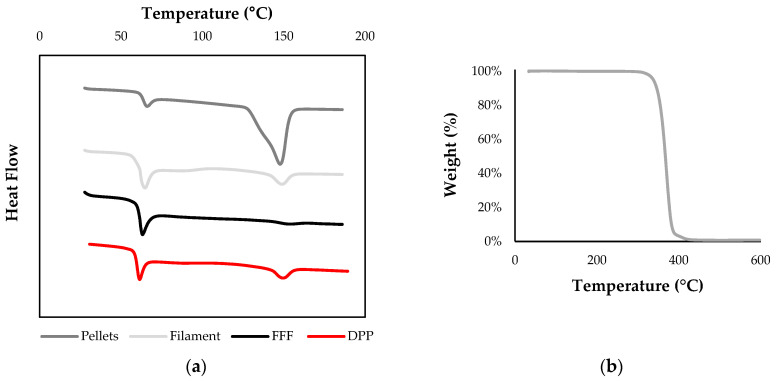
(**a**) Differential scanning calorimetry (DSC) thermograms of PLGA 85:15 raw material, filament, PAM, and Ultimaker 3 3D printed constructs with a heating rate of 10 °C/min for the first heating run. (**b**) TGA thermogram of PLGA 85:15 pellets.

Thermal characterization of PLGA pellets by DSC and ATG was used to determine the optimal temperatures for extrusion and 3D printing; the melting point and degradation temperature were *T*_m_ = 149 °C and *T*_d5%_ =304 °C, respectively (Figure 5). The raw pellet displayed a large crystallinity pic compared to processed PLGA, indicating that prior to filament fabrication or 3D printing, the material was semi-crystalline with a glass transition temperature of *T*_g_ = 63 °C. The results showed a very low crystallinity rate of PLGA 3D printed constructs for both process due to a rapid cooling rate, which limits crystallization. Nevertheless, a slight endothermic pic corresponding to a crystallinity value of 3.8% was observed for DPP printed constructs, probably due to a slower cooling time inside the 3D printing machine chamber.

### 3.3. Impact of Sterilization

**Figure 6:**
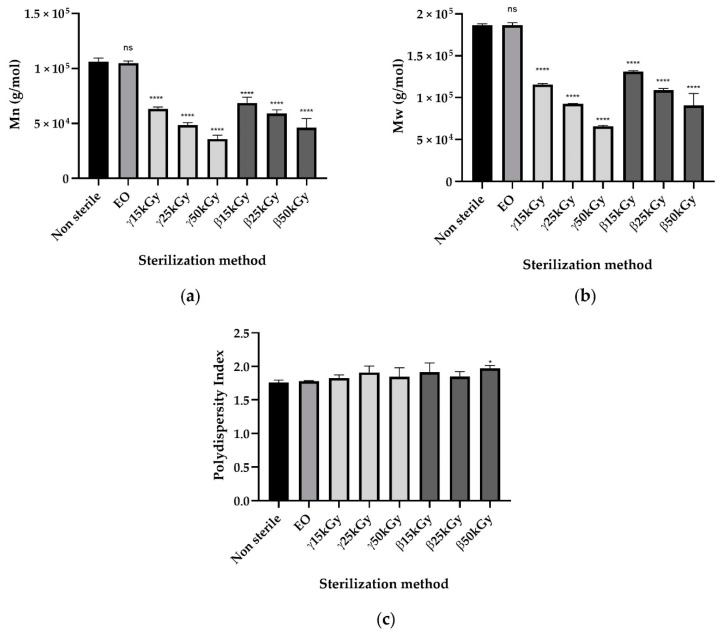
Impact of ethylene oxide as well as beta, rand, and gamma irradiation sterilization methods on the (**a**) *M*_n_, (**b**) *M*_w_, and (**c**) polydispersity index of PLGA 85:15; All data are expressed as mean ± SD (*n* = 3) and were analyzed using Anova one-way test and compared to the control * *p* < 0.05, ** *p* < 0.01, *** *p* < 0.001, **** *p* < 0.0001.

As demonstrated by the microbiological assay, sterilization of the samples was achieved effectively with each treatment gamma radiation, beta radiation, and ethylene oxide of methods because no clouding of the media was observed after 48 h of incubation time (data not shown). PLGA 85:15 samples sterilized by ethylene oxide, gamma, and beta irradiation were compared to those that were non-sterile in terms of changes in the weight average and number average molecular weight (Figure 6). For gamma and beta irradiation, all samples changed from their initial of *M*_n_ 1.062 × 10^5^ g/mol as a result of sterilization. The molecular weight data indicate that the EO sterilization processes has no significant influence on the molecular weight of PLGA 85:15 material. Gamma or beta irradiation lead to a significant drop in the initial PLGA molecular weight in a dose-dependent manner. Changes in the polydispersity index compared to the control were only statistically significant for the β50kGy group (*p* < 0.05). However, beta irradiation sterilization seems to have less impacts on PLGA molecular mass properties.

**Figure 7:**
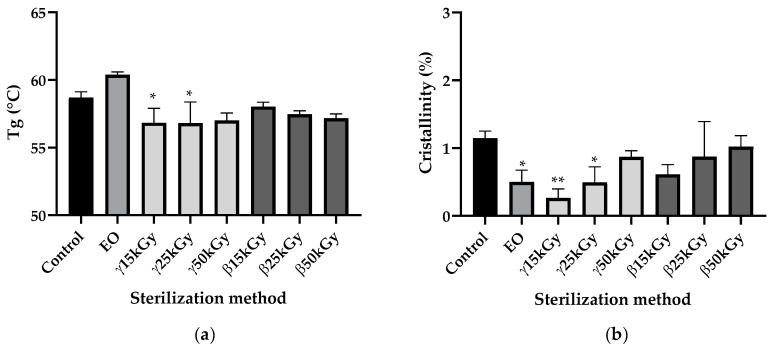
Impact of ethylene oxide, as well as beta, rand, and gamma irradiation sterilization methods on the (**a**) Glass transition temperature (*T*_g_), and (**b**) the crystallinity of PLGA 85:15; All data are expressed as mean ± SD (*n* = 3) and were analyzed using Anova one-way test and compared to control * *p* < 0.05, ** *p* < 0.01.

Scanning Differential Calorimetry (DSC) was performed to determine whether sterilization by EO, gamma, and beta irradiation could influence the crystallinity and glass transition temperature (*T*_g_) of PLGA material compared to the unsterilized starting 3D constructs (Figure 7). After EO sterilization, the *T*_g_ value of PLGA slightly increased compared to the control but the difference was not statistically different (*p* > 0.05). For gamma and beta irradiation, only the γ15kGy and γ25kGy groups showed statistically significant differences in *T*_g_ values compared to the control (*p* < 0.05), though *T*_g_ values remained far above human body temperatures (Figure 7a).

EO sterilization leads to a significant decrease in PLGA crystallinity from 1.15% to 0.50% (*p* < 0.05). For gamma and beta irradiation, only γ15kGy and γ25kGy groups show a statistically difference in crystallinity compared to the control (respectively *p* < 0.01 and *p* < 0.05); however crystallinity rates remained relatively low, so we could not conclude that ethylene oxide, gamma, and beta irradiations sterilizations have a real impact on PLGA crystallinity (Figure 7b).

### 3.4. Cytotoxicity

**Figure 8:**
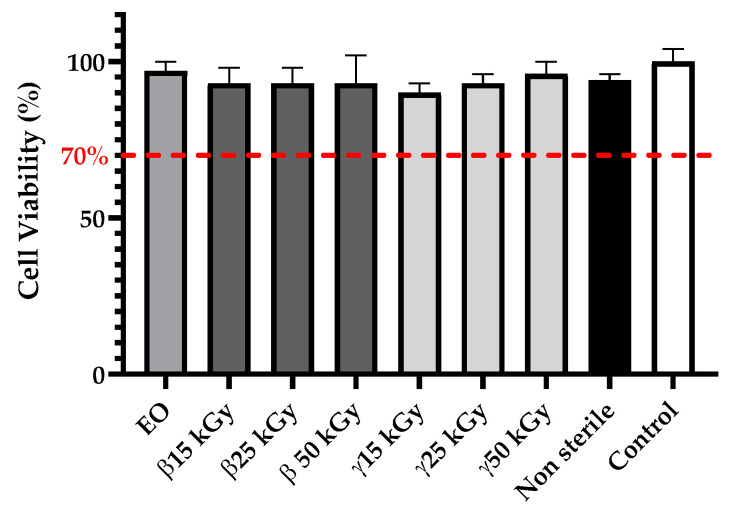
Impact of ethylene oxide, as well as beta, rand, and gamma irradiation methods on cytotoxicity of PLGA constructs after sterilization; All data are expressed as mean ± SD (*n* = 8).

Regarding the cytotoxicity assay, media extracts of PLGA samples did not affect cell viability of NIH3T3 fibroblast cells, which remained higher than 70% (Figure 8). Thus, these results demonstrated that no cytotoxic compounds are generated during EO, gamma, and beta sterilization methods.

## 4. Discussion

The aim of this study was to quantify PLGA 85:15 degradation when processed during all the production steps from additive manufacturing to terminal sterilization in order to select the production route that would have the least impact on its properties. Two additive manufacturing technologies, FFF and DPP, were compared as well as three sterilization methods: ethylene oxide, gamma irradiation, and beta irradiation. The design of the samples was chosen for the purpose of challenging 3D printers, as they require a lot of retraction and travel of print heads, thus resulting in bad printing quality if these tasks are not well managed by the device. As PLGA is sensitive to elevated heat, it likely degrades while printing; this means that printing parameters such as temperature must be adjusted to minimize degradation. Indeed, Widmer et al. have shown that PLGA’s molecular weight was decreased when the extrusion temperature was increasing [33]. These optimized printing temperatures were determined thanks to DSC and TGA analysis on pristine materials by testing different temperatures above its melting point and below its degradation temperature to achieve appropriate melt behavior. Information obtained by GPC analysis reflects how PLGA degrades during the manufacturing process. Samples printed via FFF were successively manufactured with adequate quality, but this process took longer than for DPP, as it first requires the manufacturing of the filament to occur. Furthermore, based on our experience, a large amount of polymer pellets is needed for filament extrusion, which can be an issue for research applications in particular when designing in-house material. One might assume that adding a thermal step such as filament extrusion to the manufacturing process will degrade more PLGA; however, GPC results reveals that the opposite occurred. In fact, for the PAM process, due to longer residence time of PLGA in the extrusion mechanism at elevated temperatures, raw material that wait to be processed begin to degrade progressively. Molecular weight analysis of successive printing has confirmed this hypothesis, as the *M*_n_ of PLGA decreased after each print. In a study, Shim et al. have demonstrated the impact of the residence time of PLGA at elevated temperatures on its thermal degradation by using solid free form fabrication [34]. Furthermore, to reach good printing quality by printing PLGA on PAM, printing speeds have been drastically reduced, which also increases this elongated heating period and therefore degrades PLGA. Working with a higher printing speed on DPP printer results in the under extrusion phenomenon. The Ultimaker printer enables us to increase the printing speed compared to PAM, furthermore with the FFF process, the PLGA filament is only heated punctually and during a relatively short time. Degradation generated during the manufacturing process must be considered as a bioabsorbable polymer choice for tissue engineering applications, as it impacts the resorption time announced by suppliers. Regarding printing quality, the FFF process leads to a relative smooth surface of perforated discs, whereas with the DPP process, PLGA samples show a rough top surface that could lead to undesired inflammation and discomfort after implantation in vivo. In the case of medical devices, quality control is primordial and to reach ISO 13485 certification, production must be carried out to demonstrate the ability of the process to achieve the expected results reproducibly and repeatedly. Information obtained by mass control after printing reveals that DPP printed samples has shown a large dispersity in term of mass compared to FDM printed ones and this would be an issue during process qualification because differences in mass would lead to differences in degradation behavior. Thermally-induced PLGA degradation observed in DPP printing leads to a lack of repeatability of 3D samples [35]. After an additive manufacturing step, PLGA 85:15 shows a low value of crystallinity, which is relatively interesting for bioabsorption during degradation. Indeed, as water penetration is more difficult in the crystalline area, having an amorphous polymer might reduce the chances of retaining a remaining crystalline fraction that take a longer time to degrade after the reconstruction of living tissues. As the aim of the study was to choose a manufacturing process that would minimize PLGA degradation, we would recommend for medical devices production to proceed to classic Fused Filament Fabrication in order to reach required quality specifications to be externally certified under ISO 13485. However, the DPP process should be used with more thermal stable bioabsorbable polymers, as it enables skipping the filament extrusion step and thus simplifying manufacturing process, thereby reducing the costs and qualification times that are essential for medical device development. Polycaprolactone could be a promising polymer to use with the DPP process thanks to its low melting point, thermal stability, and good melt viscosity properties [36,37,38]. Ahlinder et al. have demonstrated that the PCL molecular weight was not significantly altered after 3D printing, which makes 3D printing suitable for an additive manufacturing process that requires a longer residence time above the melting point, such as using a PAM printer. Every implantable medical device must be sterile, and sterilization is performed to eliminate viable microorganisms and thus minimize the risk of complications such as infection after implantation within the human body. For a medical device to be labeled sterile, the security assurance level (SAL) defined by the European Pharmacopeia must be SAL ≤10^−6^, meaning that the theoretical probability that a unit is nonsterile is less than one in one million. In this study, sterility testing was performed to demonstrate the absence of viable contaminating microorganisms remaining after the sterilization process. For each investigated sterilization process, a simple microbiological assay was conducted and was achieved effectively with each treatment, as no clouding of the Mueller Hinton was observed after 48 h of incubation (data not shown). However, this test only proves the sterility of the surface of the samples, as PLGA’s bioabsorbable sterility must be maintained during degradation to avoid undesirable infection complications after surgery. Indeed, even if processing PLGA 85:15 via additive manufacturing requires high temperatures that would probably kill microorganisms, the possibility cannot be excluded that still some of them could be entrapped within the layer of molten polymer and thus be released during its degradation. Furthermore, tiny crevices are present between the layer structure of a 3D printed samples where microorganisms can hide, and these parts will be more difficult to sterilize. As a key characteristic of gamma irradiation is its high penetration capacity within the bulk material, remaining viable microorganisms should be killed after performing sterilization, whereas this could be an issue for beta and ethylene oxide sterilization. Due to the limited penetration depth of electrons, sterilization via beta irradiation could be compromised for thick and complex devices; sterilization via beta irradiation should be suitable for the sterilization of 1 mm thick PLGA discs, though further study must be done to confirm our hypothesis. For ethylene oxide however, the presence of viable microorganisms within the bulk material could be a potential issue, as EO sterilization is known to be a surface sterilization. Nevertheless, the EO can penetrate some materials and can possibly sterilize the germs present in the interlayers of 3D constructs. A sterility test must be carried out during process validation to verify the destruction of the bioburden, which will allow the effectiveness of EO between layers to be confirmed. Sterilization treatments were investigated to find an effective method which is less destructive for PLGA. Gamma sterilization and beta sterilization were found to significantly decrease PLGA molecular weight, whereas after ethylene oxide treatment, PLGA’s molecular weight remains unchanged. Cleaning medical devices in order to reduce the initial bioburden before sterilization could be a solution to apply a minimal radiation dose for gamma and beta sterilization to be efficient. However, even with the smallest radiation dose tested of 15 kGy, *M*_n_ loss of PLGA was still statistically significant with 40 ± 2% and 35 ± 5% loss rates for gamma and beta irradiations, respectively (*p* < 0.0001). Faisant et al. have shown that the decrease in PLGA drug-free microparticles *M*_w_ as a function of radiation dose is almost linear and even at 4.3 kGy, degradation occurs [39]. The main issue for radiation sterilization is the polymer compatibility; indeed, these methods can degrade polymer structure because cross-linking, chain scission, or a combination of the two can occur. In our case, gamma and beta radiation induced PLGA chain scission, which result in shorter chains of macromolecules, thus leading to a significant decrease in its molecular weight. In some tissue engineering applications, this undesirable drop in PLGA molecular weight can be very problematic regarding polymer degradation, as it could lead to early loss of its properties such as its scaffold mechanical integrity and then can compromise new tissue formation. Furthermore, as beta radiation is known to have a limited depth penetration, it can cause an issue for thick devices. Ionizing rays will have more difficulties penetrating to the bulk of the device and thus can result in a heterogeneity of molecular weight within its structure. For PLGA 85:15, we would recommend using ethylene oxide sterilization, as it does not lead to chain-scission; moreover, a low-temperature ethylene oxide process enables us to sterilize PLGA devices under its glass transition temperatures, thus avoiding thermal transitions that can strongly affect mechanical properties. One of the drawbacks of the ethylene oxide method is the possible presence of EO toxic residues after sterilization such as ethylene chlorohydrin or ethylene glycol. The factors influencing the presence of these residues can be the material nature and geometry of the device to be sterilized, non-optimal aeration time, or the packaging choice. Furthermore, the inactivation of microorganisms leads to the presence of endotoxins which are components of the bacteria’s membranes and are known to be pyrogenic molecules. The use of ethylene oxide sterilization therefore requires checking the complete safety of the device during process validation according to the ISO 1135. Among all sterilization methods, the tested EO process seems to be the most suitable for PLGA 85:15; however, additional work must be done to evaluate if EO sterilization leads to changes in PLGA’s mechanical properties and morphology.

## 5. Conclusions

This work focused on finding the appropriate melt additive manufacturing route as well as sterilization process that minimize PLGA degradation. Analysis of data reveals that direct pellet printing causes high polymer degradation due to the uncontrolled residence time of PLGA in a molten state in the extrusion mechanism. This study has also shown that beta and gamma irradiation cause damages to the PLGA scaffold, while ethylene oxide sterilization is likely to have a destructive effect on PLGA molecular integrity. Based on our observations, we recommend using classic FFF printing and ethylene oxide sterilization to produce PLGA medical devices. However Direct Pellet Printing could be a promising additive manufacturing technology for more thermally stable aliphatic polyesters such as PCL. The methodology proposed in this study can be used as a guideline for the development of adequate production processes from additive manufacturing to terminal sterilization of any bioabsorbable thermoplastic polymer for medical device applications.

## Figures and Tables

**Table 1:** Manufacturing process parameters.

**PROCESS n°1**							
**Filament Extrusion Parameters**	**Zone 1** **Temperature (°C)**	**Zone 2** **Temperature (°C)**	**Zone 3** **Temperature (°C)**	**Zone 4** **Temperature (°C)**		**Extrusion speed (rpm)**	
	205 °C	210 °C	210 °C	200 °C		5 rpm	
**Printing Profile on FFF Printer**		**Printing Temperature**	**Bed Temperature**	**Printing speed**		**Flow (%)**	
		200 °C	65 °C	60 mm/s		100%	
**PROCESS n°2**							
**Printing Profile on DPP Printer**	**Cold Temperature (°C)**	**Extruder Temperature (D)**	**Head Temperature (E)**	**Bed Temperature**	**Printing speed**		**Flow**
	65 °C	170 °C	210 °C	65 °C	10 mm/s		100%

## Data Availability

No new data were created or analyzed in this study. Data sharing is not applicable to this article.

## References

[1] Franchetti M., Kress C. (2017). An Economic Analysis Comparing the Cost Feasibility of Replacing Injection Molding Processes with Emerging Additive Manufacturing Techniques. Int. J. Adv. Manuf. Technol..

[2] Nikoubashman O., Heringer S., Feher K., Brockmann M.-A., Sellhaus B., Dreser A., Kurtenbach K., Pjontek R., Jockenhövel S., Weis J. (2018). Development of a Polymer-Based Biodegradable Neurovascular Stent Prototype: A Preliminary In Vitro and In Vivo Study. Macromol. Biosci..

[3] Liao L., Peng C., Li S., Lu Z., Fan Z. (2017). Evaluation of Bioresorbable Polymers as Potential Stent Material—In Vivo Degradation Behavior and Histocompatibility. J. Appl. Polym. Sci..

[4] de Melo L.P., Salmoria G.V., Fancello E.A., de Mello Roesler C.R. (2017). Effect of Injection Molding Melt Temperatures on PLGA Craniofacial Plate Properties during In Vitro Degradation. Int. J. Biomater..

[5] Maurus P.B., Kaeding C.C. (2004). Bioabsorbable Implant Material Review. Oper. Tech. Sports Med..

[6] Konan S., Haddad F.S. (2009). A Clinical Review of Bioabsorbable Interference Screws and Their Adverse Effects in Anterior Cruciate Ligament Reconstruction Surgery. Knee.

[7] Ramot Y., Haim-Zada M., Domb A.J., Nyska A. (2016). Biocompatibility and Safety of PLA and Its Copolymers. Adv. Drug Deliv. Rev..

[8] Mohseni M., Hutmacher D.W., Castro N.J. (2018). Independent Evaluation of Medical-Grade Bioresorbable Filaments for Fused Deposition Modelling/Fused Filament Fabrication of Tissue Engineered Constructs. Polymers.

[9] Prabhu B., Karau A., Wood A., Dadsetan M., Liedtke H., DeWitt T., Li B., Webster T. (2018). Bioresorbable Materials for Orthopedic Applications (Lactide and Glycolide Based). Orthopedic Biomaterials: Progress in Biology, Manufacturing, and Industry Perspectives.

[10] Gunatillake P.A., Adhikari R. (2003). Biodegradable Synthetic Polymers for Tissue Engineering. Eur. Cell Mater..

[11] Chye Joachim Loo S., Ping Ooi C., Chiang Freddy Boey Y. (2005). Influence of Electron-Beam Radiation on the Hydrolytic Degradation Behaviour of Poly(Lactide-Co-Glycolide) (PLGA). Biomaterials.

[12] Woodard L.N., Grunlan M.A. (2018). Hydrolytic Degradation and Erosion of Polyester Biomaterials. ACS Macro Lett..

[13] Ahlinder A., Fuoco T., Finne-Wistrand A. (2018). Medical Grade Polylactide, Copolyesters and Polydioxanone: Rheological Properties and Melt Stability. Polym. Test..

[14] Ahlinder A., Fuoco T., Morales-López Á., Yassin M.A., Mustafa K., Finne-Wistrand A. (2020). Nondegradative Additive Manufacturing of Medical Grade Copolyesters of High Molecular Weight and with Varied Elastic Response. J. Appl. Polym. Sci..

[15] Jain S., Fuoco T., Yassin M.A., Mustafa K., Wistrand A.F. (2019). Printability and Critical Insight into Polymer Properties during Direct- Extrusion Based 3D Printing of Medical Grade Polylactide and Copolyesters. Biomacromolecules.

[16] Sterilization of Implantable Polymer-Based Medical Devices: A Review. https://www-sciencedirect-com.ressources-electroniques.univ-lille.fr/science/article/pii/S0378517317311304?via%3Dihub.

[17] Sterilization Techniques for Biodegradable Scaffolds in Tissue Engineering Applications. https://www.ncbi.nlm.nih.gov/pmc/articles/PMC4874054/.

[18] Holy C.E., Cheng C., Davies J.E., Shoichet M.S. (2000). Optimizing the Sterilization of PLGA Scaffolds for Use in Tissue Engineering. Biomaterials.

[19] Haim Zada M., Kumar A., Elmalak O., Mechrez G., Domb A.J. (2019). Effect of Ethylene Oxide and Gamma (γ-) Sterilization on the Properties of a PLCL Polymer Material in Balloon Implants. ACS Omega.

[20] Pietrzak W.S. (2010). Effects of Ethylene Oxide Sterilization on 82: 18 PLLA/PGA Copolymer Craniofacial Fixation Plates. J. Craniofac. Surg..

[21] Phillip E., Murthy N.S., Bolikal D., Narayanan P., Kohn J., Lavelle L., Bodnar S., Pricer K. (2013). Ethylene Oxide’s Role as a Reactive Agent during Sterilization: Effects of Polymer Composition and Device Architecture. J. Biomed. Mater. Res. Part B Appl. Biomater..

[22] Ethylene Oxide Sterilization of Medical Devices: A Review. https://www.sciencedirect.com/science/article/abs/pii/S0196655307000521.

[23] Ethylene Oxide Potential Toxicity: Expert Review of Medical Devices: Vol 5, No 3. https://www.tandfonline.com/doi/abs/10.1586/17434440.5.3.323.

[24] Lucas A.D., Merritt K., Hitchins V.M., Woods T.O., McNamee S.G., Lyle D.B., Brown S.A. (2013). Residual Ethylene Oxide in Medical Devices and Device Material. J. Biomed. Mater. Res. Part B Appl. Biomater..

[25] Kinetics of the Aeration of Ethylene-Oxide Sterilized Plastics. https://www.sciencedirect.com/science/article/pii/0142961280900381.

[26] Aeration of Ethylene Oxide-Sterilized Polymers. https://www.sciencedirect.com/science/article/pii/0142961286901080.

[27] FDA Ethylene Oxide Sterilization for Medical Devices. https://www.fda.gov/medical-devices/general-hospital-devices-and-supplies/ethylene-oxide-sterilization-medical-devices.

[28] Loo S.C.J., Ooi C.P., Boey Y.C.F. (2004). Radiation Effects on Poly (Lactide-Co-Glycolide) (PLGA) and Poly(l-Lactide) (PLLA). Polym. Degrad. Stab..

[29] Davison L., Themistou E., Buchanan F., Cunningham E. (2018). Low Temperature Gamma Sterilization of a Bioresorbable Polymer, PLGA. Radiat. Phys. Chem..

[30] Montanari L., Cilurzo F., Selmin F., Conti B., Genta I., Poletti G., Orsini F., Valvo L. (2003). Poly (Lactide-Co-Glycolide) Microspheres Containing Bupivacaine: Comparison between Gamma and Beta Irradiation Effects. J. Control. Release.

[31] Cingolani A., Casalini T., Caimi S., Klaue A., Sponchioni M., Rossi F., Perale G. (2018). A Methodologic Approach for the Selection of Bio-Resorbable Polymers in the Development of Medical Devices: The Case of Poly(l-Lactide-Co-ε-Caprolactone). Polymers.

[32] Tsuji H., Mizuno A., Ikada Y. (2000). Properties and Morphology of Poly(L-lactide). III. Effects of Initial Crystallinity on Long-term in Vitro Hydrolysis of High Molecular Weight Poly(L-lactide) Film in Phosphate-buffered Solution. J. Appl. Polym. Sci..

[33] Widmer M.S., Gupta P.K., Lu L., Meszlenyi R.K., Evans G.R.D., Brandt K., Savel T., Gurlek A., Patrick C.W., Mikos A.G. (1998). Manufacture of Porous Biodegradable Polymer Conduits by an Extrusion Process for Guided Tissue Regeneration. Biomaterials.

[34] Shim J.-H., Kim J.Y., Park J.K., Hahn S.K., Rhie J.-W., Kang S.-W., Lee S.-H., Cho D.-W. (2010). Effect of Thermal Degradation of SFF-Based PLGA Scaffolds Fabricated Using a Multi-Head Deposition System Followed by Change of Cell Growth Rate. J. Biomater. Sci. Polym. Ed..

[35] Ragaert K., Cardon L., Dekeyser A., Degrieck J. (2010). Machine Design and Processing Considerations for the 3D Plotting of Thermoplastic Scaffolds. Biofabrication.

[36] Woodruff M.A., Hutmacher D.W. (2010). The Return of a Forgotten Polymer—Polycaprolactone in the 21st Century. Prog. Polym. Sci..

[37] Soufivand A.A., Abolfathi N., Hashemi A., Lee S.J. (2020). The Effect of 3D Printing on the Morphological and Mechanical Properties of Polycaprolactone Filament and Scaffold. Polym. Adv. Technol..

[38] Peltola S.M., Melchels F.P.W., Grijpma D.W., Kellomäki M. (2008). A Review of Rapid Prototyping Techniques for Tissue Engineering Purposes. Ann. Med..

[39] Faisant N., Siepmann J., Richard J., Benoit J.P. (2003). Mathematical Modeling of Drug Release from Bioerodible Microparticles: Effect of Gamma-Irradiation. Eur. J. Pharm. Biopharm..

